# Clinical Remarks on Surgical Cases Occurring in the Buffalo Hospital of the Sisters of Charity

**Published:** 1871-02

**Authors:** J. F. Miner


### ART. VIII.—*Clinical Remarks on Surgical Cases occurring in the Buffalo Hospital of the Sisters of Charity, by* Dr. J. F. Miner.

REPORTED BY W. W. MINER, MEMBER OF THE CLASS.

Case XIII.—*Prolapsus Ani.*—We have here a case which is of double interest. The patient was a soldier during the recent war, and contracted chronic dysentery. The tenesmic efforts occasioned by this disease has resulted in prolapse of anus and rectum. He is able as you see, by voluntary effort to produce this large protrusion, which is easily reducible by hand and compress. The point in the case, which, perhaps, is of greater interest than the prolapse, is that the ulcers of the large intestine, which are occasioned in chronic dysentery or camp diarrhoea, are here visible in the rectum of a living patient. These ulcerations are of oval form, and of about three-fourths of an inch in diameter. The protruded intestine is thickened in character, and of a redder color than is natural.

The cause which first effected prolapse in this case is, therefore, evident. Chronic dysentery, which is, perhaps, the most fruitful source of prolapse, arise from some foul condition of the neighborhood or of the camp. Surgeon Woodward remarks, that the Levitical law enjoined the carrying of a paddle on the arm by every one of the Jews, and this was probably used for the covering of fecal matter. Animal instinct, also, points towards the propriety of having all animal excrement promptly rendered innocuous and removed from the possibility of infection.

Atony of the muscular parts about the region of the rectum from constitutional condition, as is found in aged persons, is one of the causes of prolapse. Again, it may be produced by violent, prolonged sympathetic or voluntary efforts at evacuation. These may be excited by abnormal conditions of any part of the large intestine, the generative or the urinary organs, or by the presence of ascarides. Both atony and excessive exertion may combine to produce this condition. In children, where prolapse is often seen, the want of curve in the direction of the sacrum, and the rectum, is made explanatory of its occurrence.

The size of the protrusion varies greatly—sometimes the mucous membrane just above the anus is alone involved. If the protruded parts become strangulated, and continue so, sloughing will naturally occur, and the tissues from which the slough separates will unite together.

If it is possible to remove the diseased condition which gives rise to prolapse, you may oftentimes thus become entirely rid of the prolapse itself. The patient may avoid tenesmic efforts, or may defecate while standing or lying. The bowels may be kept open by the administration of aperients, if desired. The protrusion is generally easily reduced in the manner already indicated. If it is found impossible to reduce a strangulated prolapse of intestines, the fibres of the constricting sphincter ani may be slit after the manner of the operation for hernia; but this is to be done with reluctance. For habitual prolapse, astringent applications to the rectum may be serviceable. Ligation of a portion of the extruded part, or removal of a V shaped portion of the anus with the scissors, are operative methods which need seldom be resorted to. The present patient is recovering from his chronic dysentery, and with its dis appearance that of the prolapse will probably be coincident.

CASE XIV.—*Club Foot.*—Talipes, or club foot, consists in a peculiar malposition of the foot, in which the bones of the foot are misplaced with reference to each other, and the whole foot is misplaced with reference to the leg above. In this affection the bones of the foot are more or less malformed; the ligaments on one side stretched, on the other shortened; the muscles attenuated; and one set of tendons is contracted while another set is lengthened. The deformity which is produced is very noticeable, though it is of quite varying degree. I believe that true club foot is always congenital. It may be approximated by causes operating after birth: it is generally believed that it may be altogether acquired, but such belief is probably unfounded: such a theory will explain only the cases of slight deviation from natural position. Accidental causes, convulsive action of muscles, fracture of bone, and luxation, may cause deformity similar to true club foot, but these are not the occasion of talipes proper.

It is said by Prof. Gross, that the etiology of club foot has never been satisfactorily explained. He says, that "the hypothesis of arrested developement, so warmly advocated by some modern pathologists, is altogether untenable, being essentially contrary to the facts of the case in every particular." Then again he says, "it must be acknowledged, however, that instances occasionally do occur, although rarely, which strongly favor the doctrine under consideration," and then cites from his own practice two cases of infants born at full term, but who died immediately after birth, who had each well marked hare-lip, cleft palate, and club foot, which he says is the result, so far as we can judge, of an arrest of development. The difficulty here, I imagine, is that while the theory of arrest of development will explain the occurrence of hare-lip and cleft palate, it is not sufficient to explain the origin of club foot. Still another theory besides arrest of development is given, which also has not been thought to sufficiently explain the causation of club foot: this is deficiency in amniotic fluid. The only objection to the theory that it alone, together with the consequent pressure of the uterus upon the foetus, produces club foot, is, that were this so, corresponding malformations would be found in the nose, chin, head, legs and knees.

The same author before quoted, in conclusion, remarks, upon the etiology of the affection, "that the most plausible view, perhaps, that can be formed in the present state of the science, of the formation of club foot, is, that it is produced by a defect of the nervous influence." In this way is explained the permanent contraction of certain muscles with corresponding malformation of bone of club foot, as well, also, a possibly co-existing atrophied and contracted state of the muscles of the back, shoulder, hand, etc. Moreover, it is said that club foot is not unfrequently associated with imperfect development of the cerebro-spinal axis and certain classes of nerves. But, after all, cases of club foot are very frequently and perhaps generally found to give no symptoms of nervous deficiency other than those which present in one ill-formed extremity.

Hare-lip and club foot do not spring from the same causes, even though associated in the same subject: at least there is a good way of according for non union of the lip and palate in the centre, which does not apply to club foot. If the toes are left off from an otherwise perfect foot, it is certainly an arrest of development, but there is no arrest in club foot. Every part of a natural foot is found in congenital club foot, and Prof. Gross' two cases show nothing for arrested development as far as club foot is concerned.

Since the treatment we are to institute in club foot will be greatly varied according to our theory of its construction, we should settle, as well as we may, this point, in order to act consistently and efficiently. It appears to me quite probable that club foot is caused by the restraint of the part involved, from its normal state of activity and exercise.

Numerous foetal specimens, which I have and will show you at the proper time, are such as to confirm in me the belief that club foot is caused by deficiency of amniotic fluid, and consequent direct pressure upon the foetus in utero. One of these specimens does indeed show deformity of the nose and frontal bone, plainly caused by the pressure of a hand also deformed, directly upon these parts. The reason that the feet are oftenest misshapen is because they present in the foetus a projecting point, which is, therefore, more easily pressed upon. It is not uncommon to find contraction of the adductor muscles of the thigh, and of the flexors of the leg, occurring at the same time, and from the same causes, with club foot. This binding down of a foot in a fixed position, by mechanical restraint, prevents motion and exercise during the whole period of intra-uterine life. Such prolonged disuse of a limb is sufficient to account for its succeeding state of atrophy and attenuation. Such a view of the causation of this affection relieves it of much of the intangibility which was consequent upon the arrest of development and faulty innervation theories.

Four principal varieties of talipes exist: these are, varus, valgus, equinus, and calcaneus; inversion, eversion, extension and flexion of the foot. In either case the tendons on the side toward which the foot projects are contracted, and those opposite them are lengthened. The method of operation is to divide by subcutaneous incision the contracted tendons, whatever they may be, which prevent the foot from being brought into its normal position. Having divided the resisting tendons, firmly bring the foot into its proper position, moulding, as it were, the bones into proper shape and place. Maintain this proper position of the foot when it has been attained, by a firm adhesive strap dressing, applied after the manner which you now observe. A shoe properly constructed—Dr. Sayre's shoe for example—may then be used as an adjunct to the adhesive dressing, but is not a substitute for it. The adhesive dressings should be reapplied once a week for some time, and great care taken in maintaining the foot in its proper place. Other and similar cases, which will be presented before you, will afford me opportunity to speak more particularly of important points in the treatment of this condition of deformity.

